# Synthesis of Keratin Nanoparticles Extracted from Human Hair through Hydrolysis with Concentrated Sulfuric Acid: Characterization and Cytotoxicity

**DOI:** 10.3390/ma17153759

**Published:** 2024-07-30

**Authors:** Otavio A. Silva, Ariane R. S. Rossin, Antônia M. de Oliveira Lima, Andressa D. Valente, Francielle P. Garcia, Celso V. Nakamura, Heveline D. M. Follmann, Rafael Silva, Alessandro F. Martins

**Affiliations:** 1 Department of Chemistry, State University of Maringa, Maringa 87020-900, PR, Brazil; pg54566@uem.br (O.A.S.); ariane.rossin@unioeste.br (A.R.S.R.); millena.lima@bolsista.ifma.edu.br (A.M.d.O.L.); hdmfollmann2@uem.br (H.D.M.F.); rsilva2@uem.br (R.S.); 2 Postgraduate Program in Chemistry, State University of West Paraná, Toledo 85903-000, PR, Brazil; 3 Research Laboratory, Federal Institute of Maranhão-Imperatriz, Imperatriz 65900-000, MA, Brazil; 4 Department of Basic Health Sciences, State University of Maringa, Maringa 87020-900, PR, Brazil; pg403857@uem.br (A.D.V.); fpgarcia2@uem.br (F.P.G.); cvnakamura@uem.br (C.V.N.); 5 Laboratory of Materials, Macromolecules, and Composites, Federal University of Technology-Paraná, Apucarana 86812-460, PR, Brazil; 6 Department of Chemistry, Pittsburg State University, Pittsburg, KS 66762, USA

**Keywords:** acid hydrolysis, human hair, nanoparticle, biomaterial

## Abstract

Human hair, composed primarily of keratin, represents a sustainable waste material suitable for various applications. Synthesizing keratin nanoparticles (KNPs) from human hair for biomedical uses is particularly attractive due to their biocompatibility. In this study, keratin was extracted from human hair using concentrated sulfuric acid as the hydrolysis agent for the first time. This process yielded KNPs in both the supernatant (KNPs-S) and precipitate (KNPs-P) phases. Characterization involved scanning electron microscopy (SEM), Fourier-transform infrared spectroscopy (FTIR), Zeta potential analysis, X-ray diffraction (XRD), and thermogravimetric analysis (TG). KNPs-S and KNPs-P exhibited average diameters of 72 ± 5 nm and 27 ± 5 nm, respectively. The hydrolysis process induced a structural rearrangement favoring β-sheet structures over α-helices in the KNPs. These nanoparticles demonstrated negative Zeta potentials across the pH spectrum. KNPs-S showed higher cytotoxicity (CC_50_ = 176.67 µg/mL) and hemolytic activity, likely due to their smaller size compared to KNPs-P (CC_50_ = 246.21 µg/mL), particularly at concentrations of 500 and 1000 µg/mL. In contrast, KNPs-P did not exhibit hemolytic activity within the tested concentration range of 32.5 to 1000 µg/mL. Both KNPs demonstrated cytocompatibility with fibroblast cells in a dose-dependent manner. Compared to other methods reported in the literature and despite requiring careful washing and neutralization steps, sulfuric acid hydrolysis proved effective, rapid, and feasible for producing cytocompatible KNPs (biomaterials) in single-step synthesis.

## 1. Introduction

Keratin is a structural and protective protein found in mammalian tissues (e.g., hair, wool, nails, fur, skin, hooves, and horns) and avian tissues (e.g., beaks and feathers), playing a crucial role in protecting the body against injuries and infections by microorganisms [1,2,3]. It is a fibrous protein rich in cysteine, structurally divided into α-keratins (α-helix) and β-keratins (β-pleated sheets). α-Keratins are the main constituents of soft tissues such as wool and hair, while β-keratins are more rigid and are present in avian claws and beaks. Disulfide bonds form bridges between cysteine chains, and hydrogen bonds in the keratin structures confer attractive properties, such as high mechanical strength, stability, and rigidity [2,4].

Keratin is widely used to manufacture materials in various fields, such as scaffolding matrices and bandages in tissue engineering and the production of controlled drug release systems. This is mainly due to its exceptional biocompatibility properties, biodegradability, mechanical durability, biological activity (e.g., wound healing ability and control of epithelial cell growth), and natural abundance [5]. Among the most promising applications of keratin is the production of dressings that enhance the regeneration of damaged skin tissues, aiding in healing acute or chronic wounds [6,7]. This regenerative and healing capacity is due to keratin’s ability to form a three-dimensional matrix that favors cell adhesion, proliferation, and infiltration. Additionally, keratin-based materials are widely used in cosmetics, leather, and agriculture [8,9]. Keratin-based nanoparticles can be used in various applications, ranging from tissue engineering [10] to biofertilizers and biosorbents for metal ions and dye removal [11,12,13].

This versatility is due to the nanoparticles’ small dimensions, high surface area, low diffusion resistance, and good cellular penetration. Nanoparticles with an average size of approximately 50 nm were obtained using an electrospray procedure. The anticancer drug doxorubicin (DOX) was efficiently encapsulated using gelation and ionic aggregation methods [14]. Keratin-based nanoparticles have been reported in the literature as promising vehicles for drug and active ingredient delivery [2,15]. Additionally, the development of magnetic keratins in nanocomposites to address handling and isolation issues associated with keratin nanoparticles (KNPs) has also been reported [14].

Despite its abundance and wide range of applications, the extraction and dissolution of keratin are challenging processes, making it essential to seek an effective, environmentally friendly, and low-cost technique. Various strategies can be employed for keratin extraction, including hydrolysis (acidic, alkaline, or thermal), enzymatic and microbiological treatment, thermal treatments, dissolution in ionic liquids, microwave exposure, and ultrasonic solidification processes [2,14,16,17]. It is important to note that partial or complete degradation of some constituent amino acids occurs during keratin hydrolysis. For example, tryptophan can be eliminated, while asparagine and glutamine are converted into aspartic and glutamic acids, respectively. Other amino acids like cystine, cysteine, methionine, and tyrosine may undergo partial degradation. The different extraction strategies cleave the cystine disulfide bonds, creating new residues and low molecular weight proteins and peptides [14].

The extraction of keratin primarily involves the cleavage of covalent disulfide bonds and intermolecular hydrogen bond interactions, with extraction procedures generally classified into denaturation and hydrolysis. Methods involving denaturation preserve the native molecular weight distribution and amino acid composition of keratin. At the same time, acidic and alkaline hydrolysis processes allow for the isolation of a mixture of low molecular weight proteins and polypeptides, both with low sulfur content [2,5,18].

Water-soluble keratin with a molecular weight of 120 kDa was obtained through an enzymatic cascade process, in which proteolytic enzymes such as keratinases are used to hydrolyze the peptide bonds present in the keratin structure [19,20]. On the other hand, methodologies using urea have been commonly employed as a denaturing agent to increase the solubility of extracted keratin in water. This occurs because, at higher concentrations, urea can weaken hydrophobic interactions between polypeptide chains, making the extracted keratin more soluble [5,21].

The extraction of nanoparticles in suspension is preferred over obtaining nanoparticles in precipitates when the process involves ultrasonication. KNPs extracted from human hair using the ultrasonic solidification method occur in suspension. By controlling various parameters during preparation, such as keratin concentration and ultrasound conditions, the size and characteristics of the nanoparticles can be fine-tuned, producing a uniform suspension of KNPs [2].

Therefore, based on what has already been reported in the literature, this present work aims to synthesize KNPs in a single step using an innovative extraction agent, such as concentrated sulfuric acid. Another innovative aspect of this article is the characterization performed (since two types of KNPs were obtained: one fraction in suspension (obtained without the use of ultrasonication) and another precipitated) and the evaluation of the biological properties of the extracted nanoparticles, such as the investigation of cytotoxic and hemolytic activity. Cytotoxicity testing is essential for evaluating biomaterials that may come into contact with human cells and tissues. In biomedical applications, it is used to assess the potential cytotoxic effects and any possible damage or adverse reactions on healthy cells that may come into contact with the material [22]. It is important to emphasize that producing cytocompatibility nanoparticles with low hemolytic activity is fundamental in biomedical applications. The nanoparticles produced in this work, derived from human hair pre-treated with concentrated sulfuric acid, were characterized using conventional analytical techniques (FTIR, XRD, SEM, and Zeta potential).

## 2. Materials and Methods

### 2.1. Materials

Human hair samples, serving as a source of keratin, were collected from a local barbershop in Maringá (Paraná, Brazil). These samples comprised a mixture of hair from various donors, with no distinction made regarding age, sex, or ethnic group. Cellulose membranes with a 12 kDa cutoff size (Sigma-Aldrich, São Paulo, Brazil) were used for dialysis. Reagents including sodium chloride, potassium chloride, disodium phosphate, and monopotassium phosphate were also obtained from Sigma-Aldrich, São Paulo, Brazil. Additionally, other reagents such as chloroform, methanol, ethanol, and hydrochloric acid were used in this study. All the mentioned reagents (except for the human hair) were used as provided by the manufacturer without any further purification.

### 2.2. Methods

#### 2.2.1. Pre-Treatment of Human Hair

The human hair samples were washed with soap and extensively rinsed with water. Then, they were treated with ethanol/distilled water (70:30 *v*/*v*) for 6 h. Afterward, the samples were filtered to remove the excess of ethanol and dried at room temperature. After drying, the hair was immersed in a mixture of chloroform and methanol (2:1 *v*/*v*) for 24 h to remove lipids and subsequently dried at room temperature.

#### 2.2.2. Extraction and Synthesis of Keratin Nanoparticles (KNPs)

**Figure 1:**
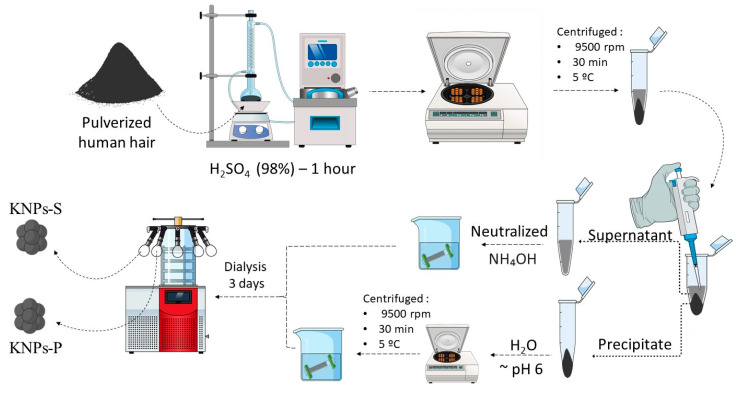
Representative scheme of KNP synthesis through hydrolysis with concentrated sulfuric acid.

The extraction and synthesis process for KNPs was developed based on the initial methodology we proposed [23], with some modifications. In a 250 mL volumetric flask, 1 g of crushed human hair and 25 mL of concentrated sulfuric acid (98% *w*/*w*) were added. The mixture was refluxed for 1 h under constant stirring and heating (100 °C). After this period, the solution was centrifuged at 9500 rpm for 30 min at 5 °C to separate the supernatant from the precipitate. The precipitate was then washed with distilled water until reaching pH 5, using the same centrifugation conditions mentioned earlier. Subsequently, the material was transferred to a cellulose membrane and dialyzed in distilled water for 3 days. During the first 6 h, the dialysis water was changed every 2 h; after that, it was changed 3 times daily. The supernatant obtained from centrifugation and separated from the precipitated material was neutralized with ammonium hydroxide (28% *v*/*v*) and transferred to a cellulose membrane, undergoing the same dialysis process used for the precipitate. Finally, both materials were frozen and lyophilized. The KNPs obtained from the lyophilization of the supernatant were named KNPs-S, while those lyophilized from the precipitates were labeled KNPs-P. The synthesis scheme of KNPs is presented in Figure 1.

### 2.3. Characterization

Fourier-transform infrared spectroscopy (FTIR) of the materials was performed using 1% *w*/*w* KBr pellets on a Bruker Vertex70 V spectrophotometer, Billerica, MA, USA. Spectra were obtained in transmission mode, covering the range from 4000 to 400 cm^−1^, with a resolution of 4 cm^−1^ and 128 scans per spectrum.

The crystallinity of the samples was analyzed by X-ray diffraction (XRD) using a Bruker^®^ X-ray diffractometer, Billerica, MA, USA, operating in the range of 4° to 80° with a resolution of 0.02° and a scanning speed of 2θ = 0.5°/min. Cu Kα radiation was generated from a source at 30 kV and 20 mA (λ = 15.406 Å).

The morphology of the samples was investigated by scanning electron microscopy (SEM) using a Thermo Scientific Scios 2 DualBeam microscope (Thermo Scientific, Waltham, MA, USA) at a voltage of 15 kV and a current of 50 μA. The KNPs were dispersed in water and applied drop by drop onto double-sided carbon tape. After drying, the samples were coated with a thin layer of gold (10 nm thick) to prevent charge accumulation. SEM images were analyzed using ImageJ 1.8.0 software (*n* = 200) to determine the average diameter of KNPs, with results expressed as mean ± standard deviation (SD).

Thermogravimetric analysis (TGA) of the samples was performed using a Perkin Elmer STA 6000 thermal analyzer, Waltham, MA, USA. Approximately 7 mg of each sample were placed in ceramic crucibles and heated from 25 °C to 800 °C at a heating rate of 10 °C/min under a nitrogen atmosphere with a 50 mL/min flow rate.

The Zeta potential (ζ) was determined using a Malvern Zetasizer Nano ZS instrument, Malvernm, UK. KNPs-S and KNPs-P (1.0 mg) were dispersed in aqueous solutions of diluted HCl or NaOH with a pH range between pH 1 and 12. For analysis, 1.5 mL (suspension samples) was transferred to a glass cell for ζ measurements in triplicate (*n* = 3) at room temperature.

### 2.4. Cytotoxicity

The cytotoxicity of the nanoparticles was evaluated against L929 fibroblast cells using the 3-(4,5-dimethylthiazol-2-yl)-2,5-diphenyltetrazolium bromide (MTT) assay [24], with modifications. Initially, KNPs were sterilized with ethylene oxide for 2 h at 40 °C (G&S Sterilization of Health Products, Maringa, Brazil) [25]. L929 cells were cultured in DMEM medium supplemented with 10% inactivated Fetal Bovine Serum (Nova Biotec, Atibaia, Brazil). For the experiment, cells were seeded at 2.5 × 10^5^ cells/mL in sterile 96-well plates and incubated for 24 h at 37 °C and 5% CO_2_ to promote cell adhesion. Subsequently, nanoparticles were seeded in wells containing L929 cells at concentrations ranging from 31.2 to 1000 µg/mL using serial dilutions. The culture plate was then incubated for 48 h at 37 °C and 5% CO_2_. Cell viability was determined using the MTT assay with an ELISA Power Wave XS spectrophotometer, Winooski, VT, USA at 570 nm. The cytotoxic concentration (CC_50_) required to inhibit 50% of L929 cells was indirectly determined using Equation (1).

(1) $$Cell\;viability\left(\%\right)=\frac{{\bar{X}}_{S}}{{\bar{X}}_{C}}\times 100$$

where ${\bar{X}}_{S}\;$ is the average absorbance of soluble formazan released from cells seeded with the samples, and ${\bar{X}}_{C}$ is the average absorbance of the negative control (untreated cells), i.e., cells in DMEM supplemented without nanoparticles. The positive control represents 100% lysed cells with the surfactant Triton X-100 (Sigma-Aldrich, São Paulo, Brazil) [26].

### 2.5. Hemolysis Assay

The hemolytic activity of the nanoparticles was evaluated according to the experimental procedure described in other studies [27,28]. A suspension of defibrinated sheep erythrocytes (520209-Laborclin, São José do Rio Preto, Brazil) at 4% was prepared in 5% glucose solution (3.09 g D (+) Anhydrous Glucose (Vetec, Verden, Germany) added to 61.8 mL distilled water). Different concentrations of nanoparticles (31.2–1000 µg/mL) were added to the glucose solution in different microtubes, followed by incubation for 3 h at 37 °C and 5% CO_2_. After this period, samples were centrifuged at 1000 rpm for 10 min. The supernatant was subjected to spectrophotometric reading at 540 nm. Positive and negative controls were obtained by substituting the test sample with 1% *v*/*v* Triton X-100 or 5% glucose. The percentage of hemolysis was determined using the absorbance values and expressed as the hemolytic concentration of 50% of erythrocytes (CH_50_), according to Equation (2).

(2) $$Hemolytic\;activity\left(\%\right)=\left(\frac{A_{pc}-A_{s}}{A_{pc}-A_{nc}}\right)\times 100$$

where $A_{pc}$, $A_{nc}$, and $A_{s}$ correspond to the absorbance values of hemoglobin from the lysed erythrocytes obtained in the positive control assays with Triton X-100, negative control assays with glucose solution without KNPs, and assays conducted in the presence of KNPs, respectively.

## 3. Results and Discussion

### 3.1. Characterization

**Figure 2:**
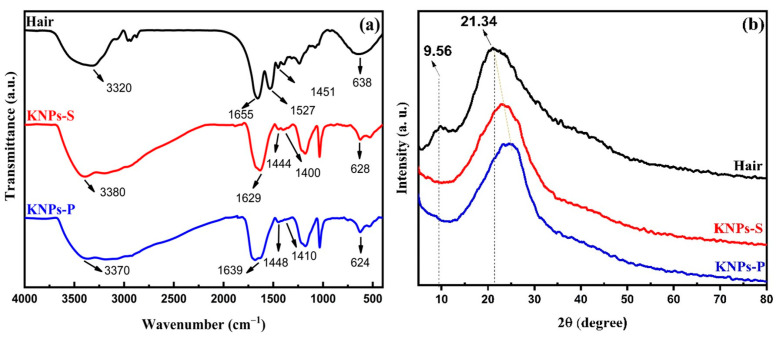
FTIR spectra (**a**) and X-ray diffractograms (**b**) of human hair (hair) before hydrolysis, keratin nanoparticles (KNPs) obtained from the supernatant resulting from hydrolysis of human hair with concentrated sulfuric acid, and keratin nanoparticles (KNPs-P) obtained from the precipitate resulting from hydrolysis of human hair with concentrated sulfuric acid.

Figure 2a shows the FTIR spectra of pulverized human hair (before hydrolysis), KNPs-S, and KNPs-P. The FTIR spectrum of human hair used in this study exhibits characteristic bands associated with keratin: the asymmetric stretching of N-H at 3320 ${cm}^{-1}$ along with the OH vibration contribution from water, the C=O stretching of amide I in α-helix structures at 1655 ${cm}^{-1}$ [23,29,30,31], and the stretching of C-N in amide II and N-H bending in the range of 1527–1451 ${cm}^{-1}$ [23,31,32]. The band at 638 ${cm}^{-1}$ corresponds to the stretching of cysteine disulfide [33,34].

The FTIR spectra of KNPs-S and KNPs-P exhibit similar band profiles compared to the bands in the FTIR spectrum of human hair. The bands at 3380 and 3370 ${cm}^{-1}$ are attributed to N-H stretching in keratin chains and the contribution of O-H vibrations from water [23,31]. Acid hydrolysis of keratin induced spectral changes in the amide I and II bands (Figure 2) due to structural changes in keratin to β-sheet and α-helix forms. Compared to pulverized human hair, the FTIR spectra of KNPs-S and KNPs-P exhibit a more pronounced band associated with the β-sheet structure at 1629 ${cm}^{-1}$ and 1639 ${cm}^{-1}$, respectively [31]. The amide II bands occur at 1444 and 1400 ${cm}^{-1}$ for KNPs-S and 1448 and 1410 ${cm}^{-1}$ for KNPs-P [31]. The same trend is observed for bands at 628 and 624 ${cm}^{-1}$ for KNPs-S and KNPs-P, respectively [33,34].

The differences in FTIR spectra between human hair and KNPs-S and KNPs-P samples likely arise from the distinct protein conformation in these materials. KNPs seem to show a more pronounced presence of β-sheet conformation compared to pulverized hair. This conformational difference may affect the rearrangement of keratin chains depending on the density of hydrogen bonds established between adjacent keratin chains. Additionally, acid hydrolysis can degrade amino acids within the keratin structure, potentially leading to altered structural conformations, and seems to favor the β-sheet conformation [35].

In a previous study by our group using concentrated hydrochloric acid (37%) for 1 h as the extraction agent, changes in keratin structure conformation were also suggested based on FTIR results, with a predominance of β-sheet keratins in KNPs synthesized [23]. Conversely, Agarwal et al. [32], who hydrolyzed human hair keratin using different agents such as sodium sulfide, peracetic acid, urea, and thioglycolic acid, and Shui-qing et al. [31], who hydrolyzed human hair keratin with a mixture of sodium hydroxide, sodium sulfite, and sodium dodecyl sulfate, observed the presence of α-helix to β-sheet structures in the synthesized KNPs. Therefore, the results seem to depend on the hydrolysis extraction agent and the experimental conditions employed.

Figure 2b presents the XRD profiles for pulverized human hair, KNPs-S, and KNPs-P samples. The hair diffractogram shows a characteristic pattern of amorphous material with ordered regions, exhibiting a broad peak in the 2θ region between approximately 15 and 40° (typical of amorphous material) and two additional peaks at 2θ = 9.5° and 2θ = 21.4°, attributed to ordered regions characteristic of α-helix and β-sheet structures, respectively [32,33,34,35,36].

In the XRD patterns of KNPs-S and KNPs-P, the peak at 2θ = 9.5° present in the hair diffractogram disappears. Additionally, a shift of the amorphous halo, related to the β-sheet structure, towards higher 2θ angles is observed. The disappearance of this peak may be associated with the removal of ordered regions in the structure of KNPs and possibly a lower content of α-helix in the extracted keratin in the KNPs compared to the native keratin in the hair.

The XRD results corroborate with previously reported data in the literature and support the FTIR data (Figure 2a). For instance, Agarwal et al. [32] observed that acid hydrolysis of human hair using peracetic acid led to the removal of ordered regions attributed to keratin in the human hair sample. Furthermore, nuclear magnetic resonance studies revealed a 10–20% reduction in the α-helix structure content of hydrolyzed keratin depending on the extraction agent used, compared to native hair. Ma et al. [37] reported similar findings, indicating that extraction of keratin mediated by urea, l-cysteine, and sodium hydroxide disrupts the semicrystalline domains of native keratin, which can be partially restored upon recrystallization depending on the hydrolysis agent. However, these ordered regions are not always re-established depending on the solvent or solvent mixtures with additives or not used for recrystallization. This effect is directly linked to the hydrolysis agent and experimental extraction conditions [38].

**Figure 3:**
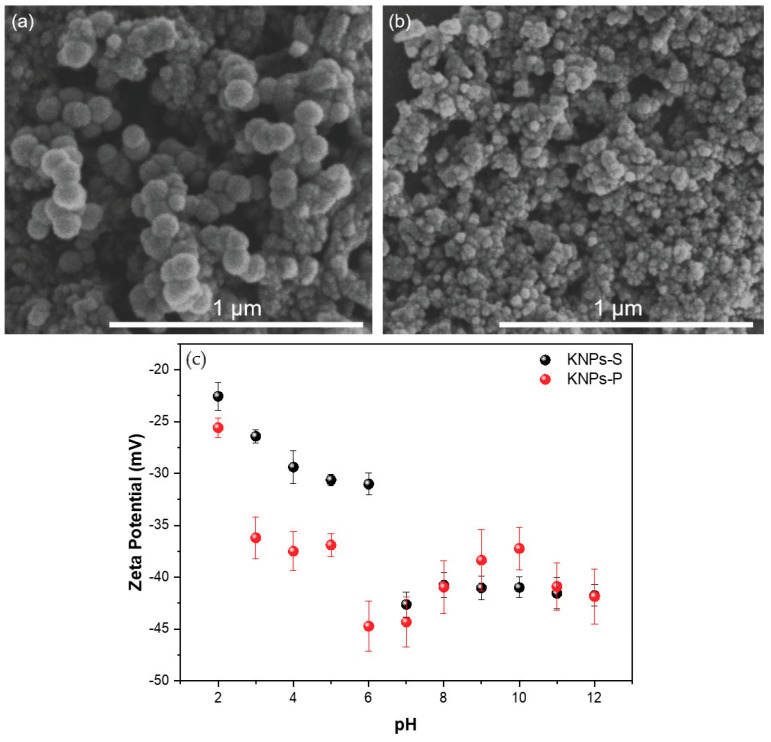
SEM images ((**a**) KNPs-P and (**b**) KNPs-S) and Zeta potentials (**c**) measured with the KNPs after lyophilization and resuspension in water in the pH range from 2 to 12. KNPs-S = keratin nanoparticles obtained from the supernatant after hydrolysis of human hair with concentrated sulfuric acid; KNPs-P = keratin nanoparticles obtained from the precipitate resulting from hydrolysis of human hair with concentrated sulfuric acid.

Figure 3 shows SEM images of KNPs-P and KNPs-S extracted from human hair samples. Both nanoparticles exhibit a well-defined spherical structure, with no significant differences in morphology and intact visual morphology. However, as expected, there is a difference in the average diameter of the nanospheres. The average diameter is 72 ± 5 nm for KNPs-P (Figure 3a) and 27 ± 5 nm for KNPs-S (Figure 3b). The difference in average sizes found in the supernatant compared to the precipitate is due to the sedimentation rate of NPs. Aggregated particles have a faster sedimentation rate and larger sizes than smaller individual particles [39].

The average diameter results are consistent with those of other studies, such as those of Abbasi et al. [40], who synthesized KNPs from human hair in a mixture of urea, thiourea, hydroxylamine hydrochloride, and 2-mercaptoethanol for 3 days at 50 °C. The nanoparticles obtained had an average size of 63.7 nm. In a previous study reported by our group [23], acid hydrolysis with concentrated hydrochloric acid (100 °C for 1 h) resulted in rod-shaped nanoparticles with average lengths and widths of 680 ± 48 nm and 190 ± 20 nm, respectively. These data indicate that the methodology applied for keratin extraction is a predominant factor influencing the morphology and average diameter of the obtained KNPs.

The spherical morphology of KNPs is associated with a smaller surface area and, consequently, lower surface energy than other nanostructures. However, the hydrolysis agent can significantly influence the morphology and geometry of the particles obtained. Studies involving cellulose extraction mediated by sulfuric acid hydrolysis have produced spherical particles [41,42].

Following neutralization, KNP-S and KNP-P aggregate despite having a negative Zeta potential below −30 mV. This aggregation is attributed to the lyophilization drying process, which promotes the clustering of suspended particles to minimize surface energy [43,44,45]. However, the precise mechanisms underlying lyophilization-induced particle aggregation are not fully elucidated [44]. It is hypothesized that lyophilization encourages the proximity of keratin chains, facilitating the formation of hydrogen bonds and van der Waals interactions, thereby promoting particle aggregation [46,47].

SEM images confirm that acidic extraction of KNPs, exposing pulverized human hair to an aqueous solution of sulfuric acid (98% *v*/*v*) for 1 h at 100 °C, was effective in obtaining keratin nanospheres in a single reaction step. Other studies show similar diameter results but with longer extraction times. For example, Abbasi et al. (2020) [40] obtained KNPs (63.7 nm) by immersing human hair in a mixture containing urea, thiourea, hydroxylamine hydrochloride, and 2-mercaptoethanol, incubated for 3 days at 50 °C. The average diameter of 63.7 nm [40] is similar to the average diameters of KNPs prepared with sulfuric acid as the hydrolysis agent. However, the reaction cycle with sulfuric acid is only 1 h at 100 °C, significantly reducing extraction time (98% saving in reaction time [40]). In addition, acid hydrolysis (1 h at 100 °C) of human hair with concentrated hydrochloric acid resulted in rod-shaped KNPs (length and width of 680 ± 48 nm and 190 ± 20 nm, respectively). KNPs extracted with hydrochloric acid have a significantly larger average size [23] than those extracted with sulfuric acid (Figure 3).

The Zeta potential (ζ) of KNPs-S and KNPs-P suspensions was measured as a function of pH (Figure 3c). Negative ζ values were observed in the investigated pH range. For KNPs-S suspensions, the values ranged from −22.56 ± 1.33 mV to −41.76 ± 3.03 mV, while for KNPs-P, the values ranged from −25.60 ± 0.92 mV to −41.87 ± 3.63 over the pH range between 2 and 12.

Extraction of KNPs with concentrated hydrochloric acid resulted in particles with positive ζ at pH below 2.8, indicating the presence of protonated amino ions (-NH_3_^+^) and hydronium (H_3_O^+^) in the suspensions. For pH values above 2.8, the Zeta potential became more negative, ranging from −10 mV to −35 mV, explained by the presence of ionized -COO− in keratin. The keratin extraction agent significantly influences the properties of the obtained KNPs, altering ζ, morphology, and particle size. Overall, ζ values become more negative with increasing pH, suggesting a predominance of carboxylate (-COO^−^), sulfate (-SO_4_^−2^), and hydroxide (-OH^−^) anions in KNP suspensions. The sulfate ions originate from sulfuric acid used as the keratin hydrolysis agent. These results are consistent with other studies. For instance, Liu et al. [48] observed a similar pattern using a urea and dithiothreitol mixture for keratin extraction (50 °C for 3 days), with ζ ranging from −15 mV (pH 4) to −25 mV (pH 10).

**Figure 4:**
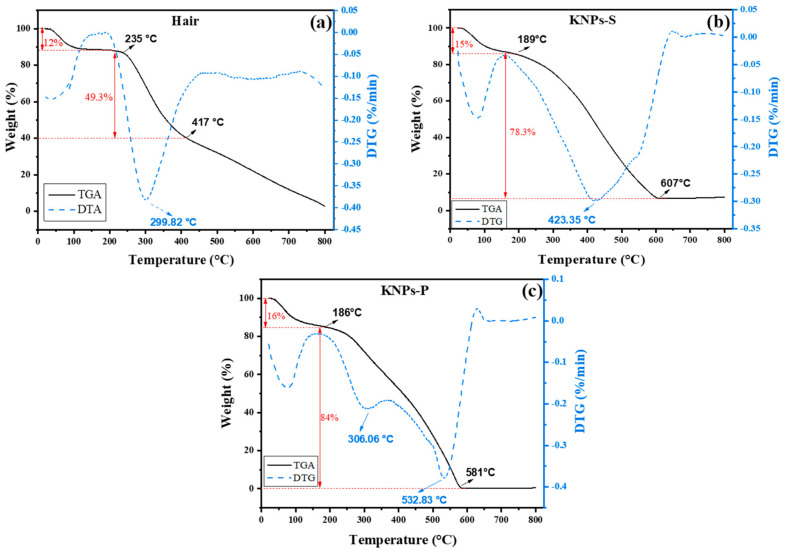
TG/DTG curves: (**a**) human hair (hair) before hydrolysis, (**b**) keratin nanoparticles (KNPs) obtained from the supernatant resulting from hydrolysis of human hair with concentrated sulfuric acid, and (**c**) keratin nanoparticles (KNPs-P) obtained from the precipitate resulting from hydrolysis of human hair with concentrated sulfuric acid.

Figure 4 presents the TG and DTG curves for hair, KNPs-S, and KNPs-P samples. The TG curve for human hair shows three distinct thermal events (Figure 4a). The first event, just below 100 °C, corresponds to the loss of water and volatile compounds. The second event, occurring in the range of 235 °C to 417 °C, is attributed to the denaturation of the keratin polypeptide chain [2]. The third event, above 418 °C, represents the complete degradation of the remaining constituents of human hair and keratin [47]. For the DTG curve of human hair (Figure 4a), three thermal events are observed: the first corresponds to the elimination of water from the structure between 50 and 100 °C, the second is centered at 299 °C, corresponding to keratin degradation [49], and the third, less pronounced event, occurs due to the oxidation of organic matter in the hair starting from 450 °C [50].

Different thermal events are observed in the TG curves of KNPs-S (Figure 4b) and KNPs-P (Figure 4c). Initially, protein denaturation occurs at lower temperatures compared to pulverized human hair. This change can be explained by the decrease in α-helix content and crystalline regions in KNPs compared to native hair, corroborating the FTIR and XRD data (Figure 2). Similar results were observed by Alahyaribeik and Ullah [51], who demonstrated that the reduced crystallinity of keratin extracted from chicken feathers results in lower thermal stability than the native keratin source material. Wang et al. [17] highlighted that the keratin extraction process decreases intermolecular interactions between keratin chains (mainly hydrogen bonds), resulting in reduced thermal stability.

The mass change was more pronounced for KNPs (78.3% for KNPs-S and 84% for KNPs-P) up to 190 °C compared to the mass change of human hair, which showed a 47% mass reduction at 235 °C (Figure 4). Agarwal et al. [32] reported this same thermal behavior with KNPs extracted by peracetic acid, attributing the result to a higher proportion of β-keratin in the obtained sample. Again, the results are consistent with the XRD data (Figure 2b). For KNPs-S and KNPs-P, the thermal event related to the evaporation of water and volatile compounds is more pronounced in the KNP TG curves than in the hair TG curve (Figure 4). This is attributed to hydrolysis, which induces a higher percentage of water and volatile compounds: 12% (hair TG curve), 15% (KNPs-S TG curve), and 16% (KNPs-P TG curve).

### 3.2. Cytotoxicity

The cytotoxicity of KNPs-S and KNPs-P was evaluated against L929 cells using the MTT method. The CC_50_ values, which represent the concentration needed to reduce cell viability by 50% compared to the control (cells not incubated with the samples, only with the culture medium), are 246.21 ± 44.8 µg/mL for KNPs-P and 176.67 ± 68.2 µg/mL for KNPs-S (Table 1).

These results indicate that the synthesized KNPs exhibited low toxicity within the concentration range evaluated, as, on average, a concentration of approximately 246 µg/mL for KNPs-P and 176 µg/mL for KNPs-S is required to inhibit 50% of cell viability. Therefore, depending on the dose, KNPs-S and KNPs-P can be considered non-toxic and suitable for biomedical applications [23,52]. Doses below 100 µg/mL are recommended. This study’s cytotoxicity results agree with those reported by Kong et al. [53].

The cytotoxicity of nanoparticles likely depends on various factors, such as composition, size, surface properties, etc., and may require case-by-case evaluation. Therefore, studies do not cite an acceptable CC50 limit indicating low nanoparticle cytotoxicity in suspension [53]. The documents suggest that the toxicity of nanoparticles is dependent on factors like size and composition, in addition to concentration, with size being a significant factor, as nanoparticles in the range of 8 to 37 nm induce greater toxicity than nanoparticles in the range of 100 nm, keeping composition and concentration fixed [54,55]. The cytotoxicity results also depend on the cell line and the type of assay used [56].

### 3.3. Hemolytic Activity

The hemolytic activity of a biomaterial is an essential criterion for evaluating its intravenous applicability or integration into devices that require contact with blood [57]. The hemolysis assay assesses the cytotoxicity of the biomaterial against red blood cells (erythrocytes). Hemolysis, or the rupture of the erythrocyte cell structure, leads to the release of hemoglobin, which is quantified spectrophotometrically at 540 nm [58]. Hemolytic activity above 5% indicates that the material is toxic to erythrocytes [57].

The HC50 of KNPs is higher than 1000 µg/mL (Table 1). Thus, the samples’ hemolytic activity (%) is presented in Table 2. KNPs-S exhibit hemolytic activity on erythrocytes at concentrations of 500 and 1000 µg/mL, whereas KNPs-P do not, as hemolysis exceeds 5% for erythrocytes treated with KNPs-S at these concentrations (Table 2). The higher cytotoxicity of KNPs-S towards erythrocytes may be related to their smaller average diameter in suspension compared to KNPs-P. The smaller size of the particles may favor their interaction with the plasma membrane, facilitating particle internalization, which consequently induces hemolysis [59]. The hemolysis results are opposite to the cytotoxicity results with fibroblast cells, as there was no statistical difference between the cytotoxicity results via MTT. This suggests that cytotoxicity results depend on the assay and the type of cells used.

The results of this study are consistent with other data reported in the literature. For example, Lu et al. [60] synthesized KNPs from human hair using urea and dithiothreitol solutions and found that the KNPs (180 nm) in suspensions of 12.5, 25, and 50 mg/mL exhibited hemolytic activity of 1.80% in rabbit blood erythrocytes, indicating blood compatibility and low hemolytic activity [57]. Yuan and collaborators synthesized KNPs incorporated with doxorubicin and tested the hemolytic activity of the material on rabbit erythrocytes in the concentration range of 10 to 100 µg/mL. The material demonstrated compatibility with erythrocytes within this concentration range [61]. Zhi et al. [62] evaluated the hemolytic activity of keratin extracted from human hair using 1-butyl-3-methylimidazolium chloride as a hydrolysis agent at 175 °C, followed by treatment with hydrochloric acid (1 M), and showed a cytocompatible material against erythrocytes with hemolysis below 1.8 ± 0.5%.

The results, including some extraction conditions for human hair-derived KNPs, as well as properties of the extracted nanoparticles, such as Zeta potential, average dry size, and cytotoxicity, are compared with findings from other studies (Table 3).

In one step, keratin hydrolysis mediated with sulfuric acid effectively extracted cytocompatible KNPs from human hair. Additionally, the washing and neutralization steps of the extracted KNPs successfully removed residual acid and other possible impurities, and low molecular weight by-products were removed through dialysis. The results found here are similar or even superior to those presented in the literature (Table 3), as cytocompatible KNPs were produced in just one synthesis step.

## 4. Conclusions

This study presents a new route for extracting keratin nanoparticles (KNPs) from pulverized human hair. KNPs-S and KNPs-P were obtained by acid hydrolysis with concentrated H_2_SO_4_ (98%) and characterized by FTIR, XRD, TGA, and Zeta potential measurements. This method enabled the extraction of two new biomaterials in a single reaction step. This study confirms that the acid hydrolysis extraction method efficiently produces spherical KNPs. Spherical and cytocompatible nanoparticles with an average diameter of approximately 100 nm were synthesized. The new keratin nanoparticle synthesis strategy presented in this study shows that both larger particles (precipitates) and suspended particles (100 nm) can be obtained in a single synthesis step and separated by centrifugation, making the presented technique promising due to its good efficiency, low operational cost, and quick handling. The proposed process is more straightforward than methods presented in other studies, as it involves only one synthesis step. A disadvantage of this study is that the washing and neutralization process of the KNPs requires dialysis, which may result in residues. However, the nanoparticles demonstrated cytocompatibility against fibroblast cells and erythrocytes at concentrations below 100 and 500 µg/mL, respectively. Therefore, KNPs show potential applications as biomaterials.

## Figures and Tables

**Table 1:** Average cytotoxic concentration (CC_50_) against fibroblast cells (L929) and hemolytic concentration (HC_50_) required to hemolyze 50% of sheep erythrocytes (520209-Laborclin).

Samples	CC_50_ (µg/mL)	HC_50_ (µg/mL)
KNPs-P	246.21 ± 44.8	>1000
KNPs-S	176.67 ± 68.2	>1000

**Table 2:** Hemolytic activity against sheep erythrocytes (520209-Laborclin).

Samples	Concentration (µg/mL)	Hemolytic Activity (%)
KNPs-P	1000	3.55 ± 6.14
	500	0.64 ± 0.90
	250	0.57 ± 0.98
	125	2.34 ± 3.30
	62.5	2.34 ± 3.30
KNPs-S	1000	13.6 ± 0.42
	500	6.60 ± 3.07
	250	3.19 ± 0.73
	125	0.43 ± 9.32
	62.5	1.49 ± 2.10

**Table 3:** Data for the synthesis of KNPs and their properties found in the literature.

Extraction Agent	Temp. (°C)/Time	Zeta (mv)	Size (nm)	Cytotoxicity CC_50_ (µg/mL)	Hemolysis (%)	Ref.
H_2_SO_4_	100 °C/1 h	−25.60 ± 0.92 to −41.87 ± 3.63	KNPs-P: 72 ± 5	246.21 ± 44.8	3.55 ± 6.14	This study
H_2_SO_4_	100 °C/1 h	−22.56 ± 1.33 to −41.76 ± 3.03	KNPs-S: 27 ± 5	176.67 ± 68.2	13.6 ± 0.42	this study
Na_2_S	K1 = 40 °C/4.5 h	−19.0 ± 3.5	-	-	-	[32]
C_2_H_4_O_3_	K2 = Room temp./overnight	−24.5 ± 10.9	-	-	-	[32]
C_2_H_4_O_2_S	K3 = 37 °C/15 h	−20 ± 6	-	-	-	[32]
CH_4_N_2_O	K4 = 50 °C/3 days	−17.7 ± 4.7	-	-	-	[32]
HCl	100 °C/1 h	20.2 to −34.4	190 ± 20	>2000	-	[23]
NH_2_C(CH_2_OH)_3_, DTT, C_2_H_6_O and CH_4_N_2_O	50 °C/72 h	−34.4 ± 8.1	190–600	-	0.30 ± 0.15, 1.11 ± 1.10 and 1.80 ± 0.65%	[60]
[BMIM]Cl and HCl	175 °C/3 h	-	-	-	1.8 ± 0.5%	[63]
CH_4_N_2_O, CH_4_N_2_S, NH_2_C(CH_2_OH)_3_ and 2-mercaptoetanol	50 °C/3 days	63.7	-	-	-	[40]

H_2_SO_4_ = sulfuric acid, Na_2_S = sodium sulfide, C_2_H_4_O_3_ = peracetic acid, C_2_H_4_O_2_S = thioglycolic acid, CH_4_N_2_O = urea, HCl = hydrochloric acid, NH_2_C(CH_2_OH)_3_ = tris(hydroxymethyl) aminomethane, DTT = dithiothreitol, C_2_H_6_O = ethanol, CH_4_N_2_S = thiourea, [BMIM]Cl = 1-butyl-3-methylimidazolium chloride.

## Data Availability

The original contributions presented in the study are included in the article, further inquiries can be directed to the corresponding author.
